# Child Effortful Control Moderates the Link Between Parenting Stress and Child Parasympathetic Regulation: Interactions Across Contexts and Measures

**DOI:** 10.1002/dev.70059

**Published:** 2025-06-26

**Authors:** Aubrey B. Golden, Daniel Ewon Choe, Leah C. Hibel, Madeline R. Olwert

**Affiliations:** ^1^ Department of Human Ecology University of California, Davis Davis California USA

**Keywords:** adaptation, context‐specificity, effortful control, parasympathetic regulation, parenting stress, respiratory sinus arrhythmia, self‐regulation

## Abstract

Parenting stress—psychosocial challenges from the parental role—is strongly tied to children's self‐regulatory abilities. Although cognitive and physiological facets of self‐regulation are integrated, research on parenting stress and children's parasympathetic activity is virtually absent. Additionally, few studies have examined changes in children's parasympathetic regulation across settings with and without a parent present. This study examined whether parenting stress is differentially associated with children's parasympathetic activity, indexed by respiratory sinus arrhythmia (RSA), as a function of their effortful control (EC). We tested whether interactions varied across EC measures (parent‐reported vs. task‐assessed) and the context of children's physiology assessment (child vs. parent–child). Parents (*N* = 67, *M* = 38.01 years) and children (*N* = 70, *M* = 51.41 months) provided data during a 2‐h lab visit. Results showed that parent‐reported EC moderated the association only in the parent–child context, whereas the task‐assessed EC moderation effect was present in both contexts. However, the effect of parenting stress on child RSA at levels of task‐assessed EC differed across contexts. Parallels in patterns of findings are discussed with reference to ecological affinity and whether a similar adaptive process emerges when both cognitive and physiological self‐regulation are assessed under comparable contextual demands.

## 1 Introduction

The construction of self‐regulation has gone through substantive change over recent decades, with persistent variation in operational definitions among subfields of psychology and developmental science. Early attempts to organize the concept broadly defined self‐regulation as internal, transactional processes that support one's ability to execute goals across diverse contexts, over time (Karoly 1993). The umbrella of self‐regulatory processes has since proliferated, encompassing integrative links to a host of social, emotional, cognitive, and biological systems of development (Bell and Deater‐Deckard 2007; Bridgett et al. 2015). Advancements in self‐regulation research are partially attributable to the growing prominence of systems theory, which posits that any developmental process is influenced by the dynamic interplay of continuous interactions between the individual and their environmental context (Overton 2013; Sameroff and Mackenzie 2003; Thompson et al. 2008). Implied in this view is the central role of parents, who serve as primary figures in children's environments and provide a foundational context for their development. The recent US Surgeon General advisory on the disproportionate stress parents are experiencing points to a pressing need to understand the ways in which parenting stress is embedded within children's environmental contexts and thus the development of their self‐regulatory abilities (Office of the Surgeon General 2024). We take an initial step toward addressing this need by examining whether parenting stress interacts with preschool‐age children's cognitive self‐regulation and is associated with their physiological self‐regulation. Further, we investigate whether these interactions are contingent on the setting of assessment or measurement approach.

### 1.1 The Hierarchical Integrative Model of Self‐Regulation

Blair and Ku (2022) proposed a hierarchical integrative model of the self‐regulation system that is largely informed by the developmental psychobiological model, experiential canalization, and a neurobiological framework. The model is composed of “higher” cognitive and “lower” emotional, behavioral, physiological, and genetic components that are recursively and reciprocally interrelated. In this model, self‐regulation is both top‐down (i.e., cognitive processes that are deliberately engaged to modulate goal‐directed behavior) and bottom‐up (i.e., physiological processes that are automatically or reflexively engaged in response to an external stimulus), and is highly susceptible to environmental influences due to the interconnected processes between cognition and the stress response. Further, Blair and Ku (2022) underscore the central importance of the parent–child relationship in the development of self‐regulation, most strongly evidenced by infants’ initial reliance on caregivers to regulate (Gartstein et al. 2013) and the protective role of primary caregivers in stressful contexts (Brody et al. 2019). Building off this model of self‐regulation, we aim to examine the dynamic between top‐down effortful control (EC) and bottom‐up physiological regulation against the backdrop of parenting stress.

#### 1.1.1 Effortful Control and Physiological Regulation

According to the integrative model, the cognitive component of self‐regulation primarily encompasses executive functions and the volitional control of attention (Blair and Ku 2022). Executive functions are effortful mental processes that serve as the foundation for complex reasoning, problem‐solving, organization, and planning (Banich 2009; Deodhar and Bertenthal 2023). EC refers to an individual's innate ability to efficiently utilize executive attention, through both shifting and focusing, in order to inhibit a dominant behavioral response and activate a subdominant response to execute a goal (Liew 2012; Rothbart 2007). Importantly, EC is a dimension of temperament widely regarded as top‐down self‐regulation with considerable conceptual and empirical overlap with executive functions, particularly inhibitory control (Kim‐Spoon et al. 2019; Tiego et al. 2020). The commonality of inhibition and attentional processes between executive function and EC has fueled an ongoing debate about the extent of their intersection and distinct contribution to top‐down self‐regulation (Eisenberg 2017; Munakata and Michaelson 2021). Some researchers have suggested that discrepant nomenclature may merely reflect the divergent foci of cognitive neuroscience and temperament research wherein each construct emerged (Zhou et al. 2012). For the purposes of our study, we opt for a general definition of EC: the ability to employ top‐down control to self‐regulate (Nigg 2017, 363).

The integrative model also outlines the importance of the autonomic nervous system, made up of the sympathetic and parasympathetic branches, as part of the self‐regulatory process (Blair and Ku 2022). The sympathetic branch has a critical role in the stress response, as sympathetic nerve fibers release catecholamines (e.g., norepinephrine), which triggers the hypothalamic–pituitary–adrenal (HPA) axis into a cascade of processes that culminate in the release of glucocorticoids (e.g., cortisol; Lengua et al. 2013). Each of these hormones is a neuromodulator, significantly impacting the rate of neuronal firing and consequential pattern of neural connectivity in the cortical regions that support top‐down cognitive processes (Bruce et al. 2013). Complementary to this integrative link between the sympathetic branch and the stress response are the vagal nerves of the parasympathetic branch. The parasympathetic vagus nerve modulates the cardiac pacemaker, which regulates sympathetic and HPA stress response activity through increased vagal influence of heart rate (Porges 2007). This vagal control of the parasympathetic branch promotes calming or resting states to support social engagement, as well as cognitive and emotion regulation (Hastings et al. 2019). Although these two branches seemingly engage in contradictory processes, their dynamic activity reflects flexible coordination to adapt to complex demands within the environment. Parasympathetic regulation is commonly indexed through respiratory sinus arrhythmia (RSA), or heart rate variability in conjunction with an individual's breathing pattern (Berntson et al. 1993). Higher RSA reflects greater parasympathetic influence and, at rest, is thought to reflect children's capacity to flexibly regulate their physiological response in support of higher‐order cognitive functions (Giuliano et al. 2018). RSA augmentation (i.e., increase from rest) is generally expected in safe, neutral contexts to support calm social engagement, while RSA suppression/withdrawal (i.e., decrease from rest) is generally expected in challenging situations to support necessary orienting without activating a sympathetic response (Hastings and Kahle 2019).

Taken together, children with efficient top‐down regulation (i.e., EC or executive functions) should also have efficient bottom‐up regulation (i.e., parasympathetic regulation). This assertion is supported by empirical evidence linking children's EC/executive functions to their parasympathetic physiology (Marcovitch et al. 2010), with some authors suggesting that parasympathetic activity may serve as a biomarker of EC or overall self‐regulatory capacity (Holzman and Bridgett 2017; Sulik et al. 2015). Although stress exposure and the parent–child relationship are considered central to these regulatory processes, the extent to which parenting stress contributes to their functioning is not yet clear. Additionally, research on the salience of different stressors within a child's social context that shape self‐regulatory processes seldom addresses the integrative dynamic between top‐down and bottom‐up self‐regulation. Moreover, the context in which we assess children's physiology and variable associations with children's EC based on measurement approach has been understudied, despite its potential to provide insight into whether dysfunction within the parent–child dyad transfers to other social contexts. Each of these gaps limits our ability to inform intervention and prevention efforts aimed at positively impacting children's developmental trajectories through bolstering their self‐regulatory abilities and improving parent–child dynamics (Hanson et al. 2019).

### 1.2 Parenting Stress

Parenting stress refers to psychosocial challenges arising from the demands of the parenting role, most often experienced within parent–child interactions (Abidin 2012; Reitman et al. 2002). This construct has been primarily studied within the framework of the family stress model (Masarik and Conger 2017), which suggests parenting stress indirectly affects child cognitive outcomes through parental behavior (i.e., *spillover*; Lengua et al. 2013; Park and Johnston 2020), such as decreased sensitivity and responsiveness to child cues (Ward and Lee 2020), and through the quality of parent–child interactions (i.e., dyadic conflict and enjoyment; Crnic et al. 2005). Parenting stress in mothers and fathers also has been shown to mediate the link between poorer parent–infant bonding quality and worse executive functioning in toddlers, suggesting cascading effects beginning in infancy (de Cock et al. 2017). Additionally, greater reports of maternal parenting stress when infants were 4 months old have uniquely predicted toddlers’ poorer EC at 18 months, whereas maternal internalizing symptoms and negative affect did not (Gartstein et al. 2013). Mothers’ perceived social support also has been shown to buffer young children's EC from the negative effects of heightened parenting stress (Yıldız and Uzundağ 2024).

Despite established links between cognitive and physiological facets of self‐regulation, research on how psychosocial parenting stress is related to children's autonomic activity is virtually absent. To our knowledge, Roos et al. (2020) is the only study to explicitly examine associations between children's autonomic functioning and parenting stress. They found parental distress, a subscale of parenting stress, was associated with children's greater sympathetic reactivity to an acute laboratory stressor and subsequent poorer cognitive task performance. While Davis et al. (2017) examined how associations between parenting stress and several child adjustment problems differed depending on children's resting parasympathetic activity, the latter was treated as a physiological sensitivity factor and was neither investigated nor expected to be directly associated with parenting stress. Although research on parenting stress and children's autonomic activity is scarce, there is a substantial body of work focused on the effects of parenting behavior and related proximal stressors. For example, greater maternal life stress has been associated with lower resting parasympathetic activity among fetuses and infants, and poorer quality parent–child interactions have been associated with lower resting parasympathetic activity among young children (for a review, see Propper and Holochwost 2013). Additionally, parents’ autonomic activity is tied to their parenting behaviors (Deater‐Deckard and Sturge‐Apple 2017) and parenting stress (Parisi et al. 2024), highlighting potential connections to children's parasympathetic regulation.

While no study has yet examined parenting stress, child EC, and child parasympathetic regulation together, there is evidence that suggests that children's top‐down self‐regulatory abilities may buffer their stress‐response physiology from the effects of parent‐related stressors. Specifically, school‐age children's task‐assessed executive functioning has been shown to moderate the association between parenting stress and children's salivary cortisol, with higher parenting stress associated with lower diurnal cortisol levels among children with poorer executive function performance (Raffington et al. 2018). Similarly, observations of parent negative affect during parent–child interactions have been associated with stronger cortisol responses to a laboratory stressor among preschool‐age children with poorer EC performance (Kryski et al. 2013). These findings raise the possibility that children's cognitive self‐regulatory abilities similarly moderate associations between parenting stress and children's parasympathetic regulation. We therefore replicate the conceptual model from these two aforementioned studies, with child EC serving as a moderator of the association between parenting stress and child RSA.

### 1.3 Context of Physiology Assessment

As described by Porges’ (2007) polyvagal theory, parasympathetic regulatory processes are elicited by the demands of one's social context. Inherent in this contextual dependency is a degree of fluidity in physiological self‐regulatory processes, raising caveats about assuming linear associations with regulatory capacity in other domains. This is illustrated by mixed findings regarding the pattern of response that is most conducive to optimal top‐down cognition. For instance, preschool‐age children with either lower baseline RSA and lower salivary cortisol, or higher baseline RSA and higher salivary cortisol have shown better executive function task performance, which suggests each physiological system may compensate for the other to support cognitive self‐regulatory ability (Braren et al. 2021). Moreover, better top‐down self‐regulation in young children has been associated with RSA suppression (Kahle et al. 2018; Phillips et al. 2024) and RSA augmentation (Holochwost et al. 2024; Utendale et al. 2014) during cognitive tasks. The extent to which different executive function and EC tasks are emotionally evocative is also related to distinct parasympathetic responses in young children (Obradovic and Finch 2017; Sulik et al. 2015). While studies considering the role of the immediate context have primarily focused on these task‐specific differences, far less attention has been given to investigating differences across social settings (Darling et al. 2022). Growing evidence suggests that children's performance on cognitive tasks is modulated by the presence of others (Frick 2025), bringing into question how this may extend to children's physiological regulation. Investigating variation in children's parasympathetic regulation across social contexts may help clarify whether there are distinct relations to children's cognitive self‐regulation in similar settings.

A particularly salient aspect of the social context to consider is whether children are engaging in tasks alone or with a parent. Notably, Skowron et al. (2014) found that among preschool‐age children, lower parasympathetic activity during tasks completed with their maltreating mother uniquely predicted the poorest inhibitory control task performance, whereas their parasympathetic activity at rest or during tasks completed alone was not associated with their inhibitory control. This pattern was not observed in a comparison group of parent–child dyads with no history of maltreatment. Thus, the presence of a parent with a history of creating a stressful environment appears to have meaningful implications for associations between children's cognitive functioning and parasympathetic regulation, particularly when assessed during joint tasks. One plausible explanation is that children's repeated exposure to harsh, unsupportive behavior induces a reduction in their parasympathetic activity within a parent–child context. In a similar vein, Meyer et al. (2019) found children's amplitudes of error‐related brain activity were elevated while completing tasks in the presence of parent with a history of controlling behavior, compared to the presence of an experimenter. This prompts consideration of whether such patterns are also linked to normative stressors within the parent–child relationship. To our knowledge, no study has yet examined context‐specific associations between children's self‐regulation and the construct of parenting stress, which all parents experience to some degree, unlike perpetrating maltreatment. Addressing this gap could contribute to a broader understanding of how everyday stress within the parent–child relationship is tied to children's self‐regulatory abilities in specific contexts.

### 1.4 Effortful Control Measurement Approach

A significant concern within self‐regulation research is the modest convergent validity between performance‐based and reporter ratings of children's EC or executive function ability (Nigg 2017). Some studies have found that performance‐based measures and teacher and parent ratings load onto a single latent construct (Schmidt et al. 2022; Sulik and Obradović 2018). Kälin and Roebers (2021) also reported a single latent construct, but lower factor loadings for parent ratings, whereas Acar et al. (2019) reported performance‐based measures and observer and secondary caregiver ratings loaded onto a single construct only when parent ratings were excluded. Given that correlations between performance‐based assessments and parent ratings of children's EC are consistently small, some authors have suggested they are measuring different constructs (Toplak et al. 2013), whereas others have suggested variations merely reflect differences in children's skills across settings and contextual demands (Acar et al. 2019). That is, aspects of children's EC may differ in emotionally evocative and ecologically valid settings with their primary caregiver, captured by parent ratings, in comparison to a more novel and structured lab environment (Frick 2025). This possibility aligns with Chang et al. (2011) suggestion, after finding different patterns of EC mediation effects between parental behaviors (e.g., punishment, warmth) and children's externalizing behavior, that different measures (i.e., parent‐reported or task‐assessed) are associated with different predictors and outcomes.

### 1.5 The Current Study

While substantial research has focused on how stressors undermine facets of children's self‐regulation, there are gaps in our understanding of the role of parenting stress, the context of children's physiological assessment (child vs. parent–child), and the salience of children's EC measurement approaches (parent‐report vs. task performance). The current study aimed to address these gaps by applying the hierarchical integrative model of self‐regulation (Blair and Ku 2022) to an examination of how parenting stress is associated with preschool‐age children's dynamic top‐down cognitive and bottom‐up physiological self‐regulatory processes. Specifically, we investigated two‐way interactive effects between parenting stress and children's parent‐reported and task‐assessed EC and their association with parasympathetic activity in a dyadic and an independent context: during parent–child interaction tasks and during an independently completed EC task battery, respectively.

In alignment with polyvagal theory, we acknowledge that children's parasympathetic activity is expected to function differently across tasks that vary in their level of challenge (Porges 2007). However, our goal was to derive a generalizable measure of children's parasympathetic functioning for each distinct context, as the primary focus of the study was to compare parallel models. If we were to compare one task from the independent context with one task from the parent–child context, any observed differences could be attributed to the task itself rather than the child's context. Therefore, we averaged children's parasympathetic activity during each context's respective tasks to address the following research questions: (1) Is the interaction between children's EC and primary caregivers’ parenting stress associated with children's parasympathetic regulation? (2) Are these interactions specific to, or do they vary by, the type of EC measurement approach (i.e., parent‐reported vs. task‐assessed)? (3) Are these interactions specific to, or do they vary by, the context of children's physiology assessment (i.e., dyadic vs. independent tasks)?

Given the vast literature on bidirectional effects between parent and child behaviors (Paschall and Mastergeorge 2016), and dynamism between cognitive and physiological processes (Blair and Raver 2012), we make no claims of unidirectional relations or causality based on the current analyses. Rather, we hypothesize the association between parenting stress and children's RSA (i.e., parasympathetic regulation) will differ based on children's EC, exemplifying adaptive top‐down regulation of bottom‐up physiological activity. That is, we expect parenting stress will be negatively associated with child RSA for children with lower EC but will have little or no association with RSA for children with higher EC. We do not hypothesize a main effect of parenting stress, as our focus is on the moderating role of children's EC, and interpreting main effects in the presence of an interaction may not be meaningful or accurate (Baron and Kenny 1986; Hayes 2017). Considering the novelty of the current study, we make no hypotheses regarding the specificity or strength of associations based on the type of EC measure or context of children's physiology assessment. However, based on prior work suggesting children's physiological and neural responses are sensitive to the presence of a parent with a history of stress‐inducing behavior (Meyer et al. 2019; Skowron et al. 2014), interactions may be specific to the dyadic, as opposed to the independent, context. Additionally, if the two EC measures reflect true differences in children's abilities across distinct settings, parent‐reported EC may be a stronger moderator for child RSA during dyadic tasks, whereas task‐assessed EC may be stronger for child RSA during independent tasks. We also generally expect parenting stress will be negatively correlated with children's EC (Gartstein et al. 2013) and RSA, and children's EC will be positively correlated with their RSA (Sulik et al. 2015).

## 2 Method

### 2.1 Participants

Sixty‐seven parents^1^ (*M* = 38.01 years, *SD* = 4.09, 88.1% mothers) and 70 children (*M* = 51.41 months, *SD* = 4.93, 48.6% girls) participated in this study. Families were recruited from local events, preschools, childcare centers, and community organizations in a suburban area adjacent to a large university. Most parents (91.0%) were married to the other biological parent of their child. Over half (52.2%) of families reported a gross household income exceeding $100,000; 20.9% reported income between $80,000 and $100,000, 7.5% between $60,000 and $80,000, 7.5% between $40,000 and $60,000, and 6.0% between $20,000 and $40,000; and 6.0% did not report their income. Most parents (58.2%) held postgraduate degrees, reflecting an overrepresentation in comparison to the city population (49.1%; US Census Bureau 2021). Among participants, 31.3% held master's degrees, 31.3% held college degrees, 26.9% held doctoral/professional degrees, 3.0% held associate's degrees, 1.5% had vocational/technical degrees, and 6.0% did not disclose their educational background. The racial–ethnic composition of the sample was representative of the city population of about 74,000 individuals. Among parents, 64.2% identified as White, 14.9% as biracial or multiracial, 13.4% as Asian or Asian American, 4.5% as other, and 3.0% as African American or Black. Additionally, 10.4% of parents identified ethnically as Hispanic/Latino.

### 2.2 Procedure

Parent–child dyads were scheduled for a 2‐h visit at an on‐campus lab space. The visit began with the research team providing study information and obtaining parent consent and child assent. Next, a researcher placed seven adhesive electrodes on the child: one on each clavicle and bottom rib, one below the sternum, and one on both the lower and upper spine. Electrodes were then plugged into a mobile ambulatory monitor where biometric data were acquired with BioLab software (MindWare Technologies, Ltd., Gahanna, Ohio). Parent–child dyads were instructed to sit on a couch and watch a 5‐min calming video to establish a baseline of physiological activity. A researcher then brought the child to a separate room to complete a battery of EC tasks in a predetermined order, while the parent was administered tasks and questionnaires. Prior to each EC task, the researcher provided instructions and practice trials to ensure the child's understanding. When tasks involved multiple scored trials, children were given rule reminders at the midpoint of scored trials. The child was offered breaks between tasks to mitigate fatigue. The task battery was video recorded for later scoring of child performance.

Once the child completed the task battery, they were reunited with their parent. Parent–child dyads were then asked to engage in four cooperative tasks: free play, clean‐up, and two Etch‐A‐Sketch games. For *free play*, a researcher instructed the parent and child to sit on the floor and play with one another as they normally would at home using age‐appropriate toys (i.e., kitchen toys, animal figurines, Mr. Potato Head, a doll house). The researcher then left the room and returned 5 min later. *Clean‐up* began upon the researcher's return, wherein the parent was instructed to guide and encourage the child to put all of the toys back into a bin without physically helping (i.e., using only his or her words to elicit the child's compliance). The researcher again left the room and returned 3 min later, or when the task was completed. Upon return, the researcher instructed the parent and child to sit next to each other on the floor at a coffee table to play together with an *Etch‐A‐Sketch* drawing toy. The parent and child were each assigned one dial on the toy and told to not use the other's dial to foster collaboration. In the first game, dyads were told to draw a pair of rectangles for 5 min. In the second game, the task difficulty increased to drawing a more complex image of a house together for 5 min. The researcher left the room during each drawing period.

The visit concluded with the child receiving a small toy and the parent receiving compensation for participation. This study was approved by the institutional review board following American Psychological Association (APA) ethical standards. This study was not preregistered.

### 2.3 Measures

#### 2.3.1 Parenting Stress

Parents completed the 36‐item Parenting Stress Index—Short Form (PSI‐SF), based on the original 120‐item questionnaire (Abidin 1990). The PSI‐SF is composed of three subscales that operationalize facets of stress within the parent–child relationship for children aged 0–12: Difficult Child (12 items; e.g., “My child seems to cry or fuss more often than most children”), Parental Distress (12 items; e.g., “I often feel I cannot handle things well”), and Parent–Child Dysfunctional Interactions (12 items; e.g., “My child rarely does things for me that make me feel good”; Abidin 2012; Lee et al. 2016). Parents rated each item on a 5‐point scale (1 = *strongly agree*, 5 = *strongly disagree*). All items were summed to create a total stress score, ranging from 36 to 180 (α = 0.81, *M* = 73.31, *SD* = 19.99; Lange et al. 2019; Reitman et al. 2002).

#### 2.3.2 Child Effortful Control

Children's EC was assessed through the laboratory task battery and a parent‐report questionnaire. The task battery consisted of 11 tasks adapted from Kochanska's (2015) Family Study EC Batteries (Kochanska et al. 1996). Three tasks were not included in the analyses due to apparent ceiling effects, wherein half of the children reached the maximum score (i.e., Whisper, Dinky Toys, and Tongue). Thus, the resulting tasks were: Day/Night (α = 0.73), Happy/Sad (α = 0.77), Bird/Dragon (α = 0.72), Tower (α = 0.46), Walk‐A‐Line Slowly (α = 0.77), Turtle/Rabbit (α = 0.53), Gift Wrap (α = 0.81), and Gift Delay (α = 0.72). Children's performance scores on the eight tasks were standardized and then averaged to create a total EC task battery score (α = 0.63; Gerstadt et al. 1994; Kochanska 2015; Kochanska et al. 1996; Lagattuta et al. 2011).

Parents completed the 94‐item Children's Behavior Questionnaire—Short Form (CBQ‐SF; Putnam and Rothbart 2006). The CBQ‐SF measures temperament qualities for children aged 3–8 years on a 7‐point scale (1 = *extremely untrue*, 7 = *extremely true*). An EC factor score (α = 0.65, *M* = 5.38, *SD* = 0.54) was calculated by averaging the Attentional Focusing (six items), Inhibitory Control (six items), Perceptual Sensitivity (six items), and Low‐Intensity Pleasure (eight items) factors. These four subscales constituted the original higher‐order factor that Rothbart et al. (2001) first identified and labeled EC, which was subsequently validated in the CBQ‐SF (Putnam and Rothbart 2006).

#### 2.3.3 Parasympathetic Activity

Children's parasympathetic activity was acquired continuously with a mobile ambulatory monitor (MindWare Technologies, Ltd., Gahanna, Ohio) during the EC task battery and parent–child interaction tasks. Electrocardiogram (ECG) data were edited by trained research assistants offline using MindWare Heart Rate Variability (HRV 3.2.4) analysis software. Waveforms within 30‐s epochs were visually inspected to remove artifacts, correct inverted ECG signals, and manually insert mid‐beats for R waves inadequately marked by the R peak detection algorithm. Data were detrended by a first‐order polynomial, cosine tapered, subjected to a Fast Fourier Transform, and used to derive a frequency domain measure of RSA from the natural log of the high‐frequency/RSA band (0.24–1.04 Hz; Berntson et al. 2017). Epochs that required editing R waves in excess of 10% of the segment were excluded from analyses. Only 30‐s epochs in which children performed the task (i.e., excluding initial instructions and practice trials, if applicable) were included in individual task RSA scores, with a total duration of 10.75 min on average (*SD* = 2.04 min, range = 6–18 min). The resulting 30‐s epoch RSA values were first averaged for each task to create indices of children's average parasympathetic activity. Children's average RSA during each task was plotted to inspect general trends in the pattern of change for both contexts. As expected, child RSA fluctuated across each context's respective tasks, and patterns of change were largely consistent across children (see Figure S1).

A series of paired‐samples *t*‐tests were conducted to examine whether RSA significantly changed from one task to the next. Across the four parent–child interaction tasks, children's RSA significantly decreased from free play (*M* = 5.16, *SD* = 0.94) to clean‐up (*M* = 4.78, *SD* = 0.82), *t*(45) = 3.87, *p* < 0.001, and increased from clean‐up to the first Etch‐A‐Sketch task (*M* = 5.44, *SD* = 1.00), *t*(45) = −5.74, *p* < 0.001, but there was no significant change from the first to the second Etch‐A‐Sketch task (*M* = 5.48, *SD* = 0.93), *t*(45) = −0.60, *p* = 0.549. Across the independent EC task battery, children's RSA did not significantly change from Day/Night (*M* = 6.12, *SD* = 1.11) to Happy/Sad (*M* = 6.03, *SD* = 1.23), *t*(44) = 0.64, *p* = 0.528, or from Happy/Sad to Bird/Dragon (*M* = 6.09, *SD* = 1.06), *t*(44) = −0.53, *p* = 0.602, but there was a significant decrease from Bird/Dragon to Tower (*M* = 5.64, *SD* = 1.14), *t*(44) = 4.15, *p* < 0.001, and from Tower to Walk‐A‐Line Slowly (*M* = 4.80, *SD* = 1.14), *t*(44) = 7.72, *p* < 0.001. There was a significant increase from Walk‐A‐Line Slowly to Turtle/Rabbit (*M* = 5.59, *SD* = 1.00), *t*(44) = 6.59, *p* < 0.001, but no change from Turtle/Rabbit to Gift Wrap (*M* = 5.73, *SD* = 1.10), *t*(44) = 1.11, *p* = 0.274, or from Gift Wrap to Gift Delay (*M* = 5.79, *SD* = 1.09), *t*(44) = 0.43, *p* = 0.667.

Next, the 30‐s epoch RSA values corresponding to each individual task were averaged to create an index of children's average parasympathetic activity across the entire EC task battery (α = 0.94, *M* = 5.14, *SD* = 0.95) and across all four parent–child interaction tasks (α = 0.91, *M* = 5.24, *SD* = 0.87). Fifteen children did not provide psychophysiological data because of discomfort or noncompliance. A binary variable was created to examine potential differences in demographic and study variables between children with and without psychophysiological data. Welch's two‐sample *t*‐test was used to examine differences among continuous variables, and Fisher's exact test was used to assess differences among categorical variables. Children with psychophysiological data (*M* = −0.11, *SD* = 1.03, *n* = 55) performed worse on the EC tasks than children without these data (*M* = 0.40, *SD* = 0.78, *n* = 15), *t*(28.70) = 2.06, *p* = 0.048. There were no significant differences between children with and without psychophysiological data for parent‐reported EC, *t*(33.83) = 1.66, *p* = 0.107, parenting stress, *t*(15.87) = 1.98, *p* = 0.070, gross household income, *t*(15.04) = −1.89, *p* = 0.078, parent education (*p* = 0.187), parent gender (*p* = 1), parent race (*p* = 0.525), parent age, *t*(17.81) = −0.41, *p* = 0.685, child gender (*p* = 0.389), child race (*p* = 0.339), or child age, *t*(20.34) = 1.65, *p* = 0.115.

### 2.4 Analytic Plan

**FIGURE 1:**
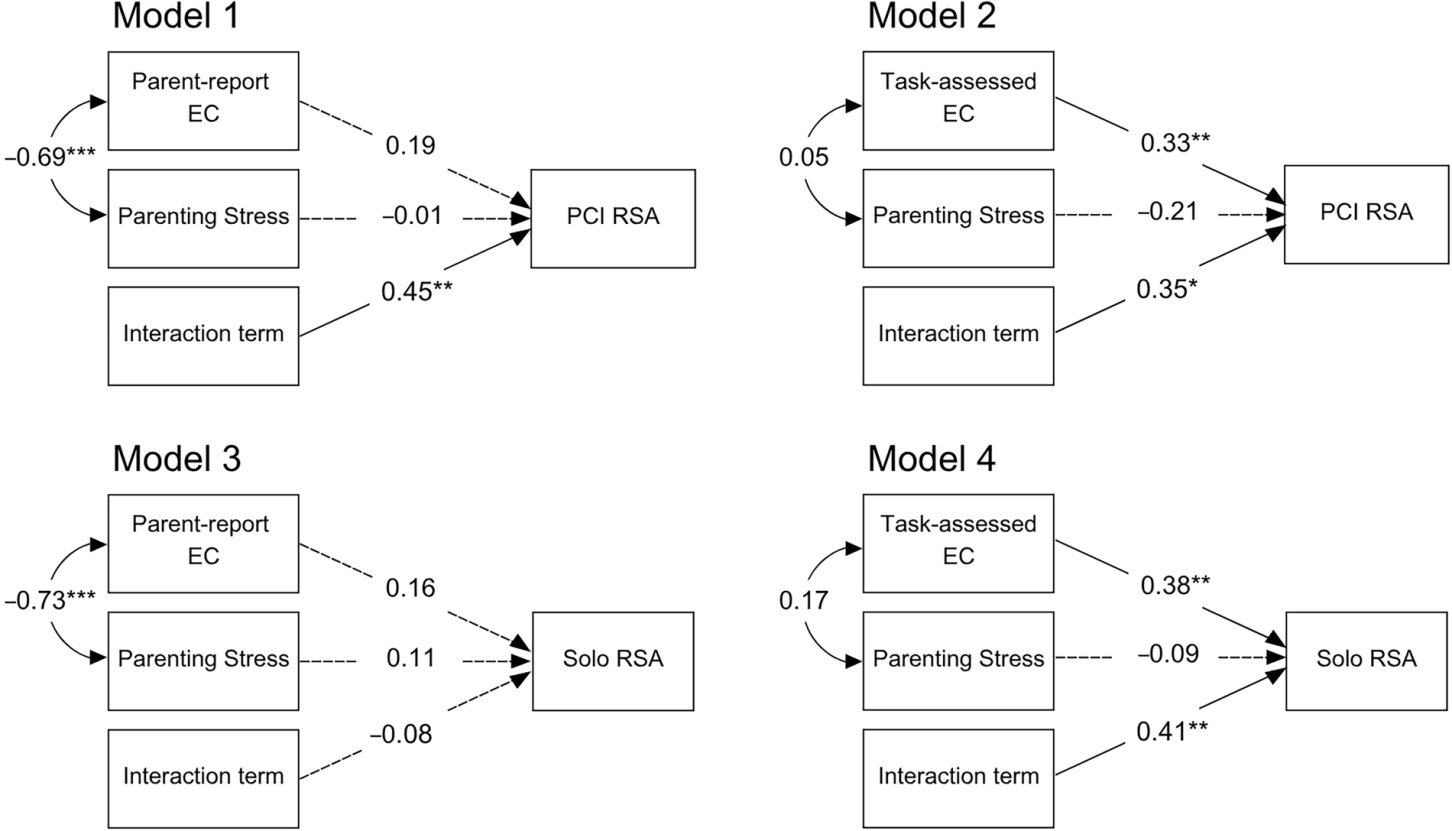
Summary of four path models testing combinations of parenting stress and child effortful control (EC) interactions and their associations with children's respiratory sinus arrhythmia (RSA) during parent–child interaction (PCI) tasks and tasks completed independently (Solo). All estimates are depicted with standardized coefficients. Model 3 depicts a re‐specified model with freely estimated parameters to address poor fit. Covariation is depicted with curved double‐headed arrows. Solid single‐headed arrows indicate statistical significance. Dashed single‐headed arrows indicate nonsignificance. *p < 0.05; **p < 0.01; ***p < 0.001.

Analyses were performed in RStudio (Posit Team, 2025). Paired‐samples *t*‐tests were conducted to examine differences in children's EC and physiology assessments. Mardia's tests of multivariate kurtosis and skewness were conducted to assess normality. Following preliminary analyses, path analysis was used to test four models, reflecting all possible combinations of the two EC measures and two physiology settings (see Figure 1), using the “lavaan” package (Rosseel 2012). Since it is plausible that children who did not provide psychophysiological data have different physiological responses than those who did, the missing at random (MAR) assumption could be violated if we included the full sample. Therefore, to mitigate potential bias and avoid violating this assumption, only children with psychophysiological data were included in the path analyses.^2^ Little's (1988) missing completely at random (MCAR) test was conducted on this subset to further evaluate missingness. To address MAR data, full‐information maximum likelihood (FIML) estimation was used (Satorra and Bentler 1994). Nonsignificant covariation with the exogenous interaction term was pruned to assess model fit. Several model fit indices were evaluated, including a robust chi‐square test of exact fit (Satorra and Bentler 2010), comparative fit index (CFI; >0.90 = good fit; Hu and Bentler 1999), root mean square error of approximation (RMSEA; <0.08 = good fit; MacCallum et al. 1996), and standardized root mean square residual (SRMR; <0.08 = good fit; Hu and Bentler 1999). Additionally, RMSEA tests of close fit were conducted to assess model misfit (null hypothesis: RMSEA ≤ 0.05). Each interaction was probed and plotted using the Johnson–Neyman technique (Johnson and Fay 1950) conducted with the “modsem” package (Slupphaug et al. 2024). This approach considers the entire distribution of the moderator and accounts for the associated standard errors and confidence intervals for each conditional effect (Hayes 2017). The result is a precise interval that identifies the region where the effect of an independent variable on the dependent variable is statistically significant, along with the corresponding slope estimates.

## 3 Results

Descriptive statistics and two‐tailed bivariate correlations are depicted in Table 1. Consistent with previous findings (Olson et al. 2017), analyses showed gender differences in EC. Girls (*M* = 5.63, *SD* = 0.42, *n* = 33) had higher parent‐reported EC than boys (*M* = 5.14, *SD* = 0.54, *n* = 34), *t*(62) = −4.14, *p* < 0.001. Similarly, girls (*M* = 0.23, *SD* = 0.85, *n* = 34) had slightly higher task‐assessed EC than boys (*M* = −0.22, *SD* = 1.09, *n* = 36), *t*(66) = −1.89, *p* = 0.031. The two EC measures showed no association, *r*(65) = 0.17, *p* = 0.164, while the two child RSA variables showed a highly positive correlation, *r*(50) = 0.86, *p* < 0.001. Parent‐reported child EC was not associated with child RSA during the parent–child interaction tasks, *r*(47) = 0.23, *p* = 0.101, or with child RSA during the independently completed tasks, *r*(50) = 0.06, *p* = 0.654. In contrast, task‐assessed EC was positively correlated with child RSA during both the parent–child interaction tasks, *r*(50) = 0.33, *p* = 0.019, and the independently completed tasks, *r*(53) = 0.40, *p* = 0.003. Parenting stress was negatively correlated with parent‐reported child EC, *r*(57) = −0.56, *p* < 0.001, but had no association with task‐assessed child EC, *r*(58) = 0.13, *p* = 0.313. Additionally, parenting stress showed a marginally significant negative correlation with child RSA during the parent–child interaction tasks, *r*(43) = −0.27, *p* = 0.073, but had no association with child RSA during the independently completed tasks, *r*(46) = −0.03, *p* = 0.847. Household income showed a positive association with parent age, *r*(64) = 0.25, *p* = 0.042, and parent education, *r*(64) = 0.30, *p* = 0.015, but there were no other significant associations among sociodemographic variables.

**TABLE 1:** Bivariate correlations and descriptive statistics of study variables.

Variable	*N*	*M* (*SD*)	1	2	3	4	5	6	7	8	9
Child age (months)	70	51.41 (4.93)	—	−0.04	0.03	−0.15	0.19	−0.04	0.20	−0.03	0.20
Parent age (years)	67	38.01 (4.09)		—	0.11	0.25^*^	−0.05	−0.03	−0.08	−0.07	−0.12
Parent education	66	6.82 (1.05)			—	0.30^*^	−0.12	0.05	0.14	−0.22	−0.17
Household income	66	5.17 (1.22)				—	−0.05	−0.18	−0.10	−0.20	−0.14
Parenting stress	60	73.31 (19.99)					—	−0.56^***^	0.13	**−0.27^+^**	−0.03
Parent‐reported EC	67	5.38 (0.54)						—	0.17	0.23	0.06
Task‐assessed EC	70	0.00 (1.00)							—	0.33^*^	0.40^**^
RSA (dyadic)	52	5.24 (0.86)								—	0.86^***^
RSA (independent)	55	5.71 (0.95)									—

*Note:* Dyadic refers to children's RSA during parent–child interaction tasks, while independent refers to children's RSA during tasks completed independently. Task‐assessed EC was standardized. Bold values are statistically significant.

Abbreviations: EC, child effortful control; RSA, respiratory sinus arrhythmia.

+*p* < 0.10; *p < 0.05; **p < 0.01; ***p < 0.001.

Paired‐samples *t*‐tests indicated no statistically significant difference between the two EC measures, *t*(66) = −0.47, *p* = 0.637, but child RSA decreased significantly from the independent tasks (*M* = 5.71, *SD* = 0.95, *n* = 55) to the parent–child interaction tasks (*M* = 5.24, *SD* = 0.86, *n* = 52), *t*(51) = −6.91, *p* < 0.001. Mardia's tests did not indicate the presence of multivariate skewness or kurtosis in the data. Since there was no evidence of normality assumption violations, results of the path model estimates are presented without robust corrections. Little's MCAR test conducted on the subset of children with psychophysiological data indicated that the data were missing completely at random, 𝜒^2^(19) = 22.90, *p* = 0.243, supporting the use of FIML to handle missing data. Among this subset, seven cases were missing parenting stress and three were missing parent‐reported EC. Gender was initially included as a covariate in the path models given the associations found with both EC measures but was removed due to a lack of significant effects and worsening of model fit. No other sociodemographic variable was included as a covariate given the lack of associations found during preliminary analyses.

Parameter estimates of each path model are depicted in Figure 1, and fit statistics are depicted in Table 2. All four models estimated 12 parameters. Model 1 and Model 3 had four patterns of missing data, and Model 2 and Model 4 had two patterns of missing data. For all models with significant interaction terms, the slope of parenting stress and its significance increased as EC values deviated further from the mean. Given the plausibility that RSA could instead moderate the association between parenting stress and EC, we re‐estimated the four models with the moderator and outcome variables switched. These alternative models showed very poor fit, suggesting a misspecification of the associations among study variables (see Table S1).

**TABLE 2:** Fit statistics of four path models.

	𝜒^2^	*df*	*p*	CFI	RMSEA	SRMR
Model 1	0.29	2	0.860	1.00	0.00	0.04
Model 2	1.96	2	0.376	1.00	0.00	0.08
Model 3	5.04	2	0.080	0.87	0.17	0.11
Model 4	0.26	2	0.878	1.00	0.00	0.04

*Note:* 𝜒^2^ = robust chi‐square test of exact fit.

Abbreviations: CFI = comparative fit index; RMSEA = root mean square error of approximation; SRMR = standardized root mean square residual.

### 3.1 Parenting Stress and Child Effortful Control Interactions and Their Association With Children's Parasympathetic Regulation During Dyadic Tasks

**FIGURE 2:**
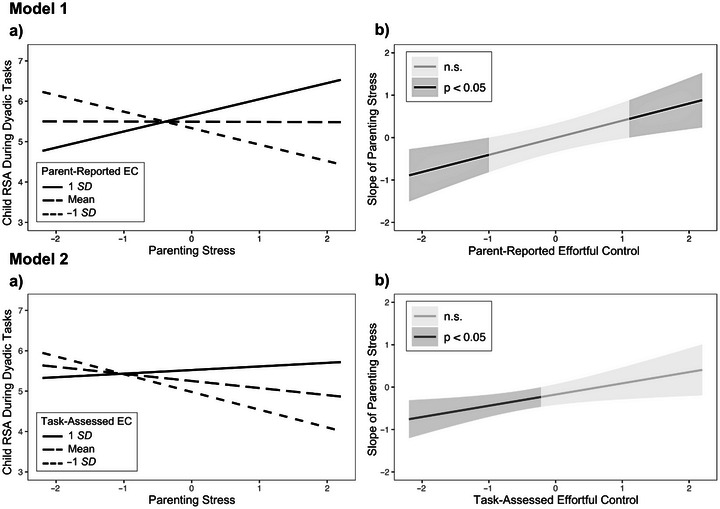
Plotted interactions (a) and regions of significance (b) for the association between parenting stress and children's respiratory sinus arrhythmia (RSA) during dyadic parent–child tasks at levels of children's parent‐reported effortful control (EC) in Model 1 and levels of task‐assessed EC in Model 2. The Johnson–Neyman regions of significance are plotted for each respective model. The slope of parenting stress reached significance (*p* < 0.05) when parent‐reported EC was outside the interval [−1.00 *SD*, +1.10 *SD*] in Model 1 and when task‐assessed EC was <0.22 *SD* below the mean in Model 2. Parenting stress and both EC measures have been standardized for interpretability. n.s. = nonsignificant.

The first model (see Model 1 in Figure 1) examined parenting stress, children's parent‐reported EC, and their interaction as independent variables and children's RSA in a dyadic context (i.e., during parent–child tasks) as the dependent variable. The model fit the data well, 𝜒^2^(2, *N* = 52) = 0.29, *p* = 0.860, CFI = 1.00, RMSEA = 0.00, SRMR = 0.04, and the null hypothesis of close fit was retained (*p* = 0.927). The independent variables accounted for 23.8% of the variance in children's RSA. Although bivariate correlations showed parenting stress was negatively and parent‐reported EC was positively correlated with child RSA during the dyadic context, neither had a significant direct effect with the interaction term included in the model. The interaction between parent‐reported EC and parenting stress was significantly associated with children's RSA during the parent–child interaction tasks (*β* = 0.45, *SE* = 0.14, *p* = 0.001). As shown in Model 1a in Figure 2, parenting stress was negatively associated with child RSA for children with lower EC and positively associated with child RSA for children with higher EC. The Johnson–Neyman analysis showed the slope of parenting stress was significant when parent‐reported EC was ≤1.00 *SD* below the mean (20.41% of cases; *β* = −0.41, *SE* = 0.20, *p* = 0.050) and when ≥1.10 *SD* above the mean (18.37% of cases; *β* = 0.44, *SE* = 0.22, *p* = 0.050; see Model 1b in Figure 2).

The second model (see Model 2 in Figure 1) examined parenting stress, children's task‐assessed EC, and their interaction as independent variables and children's RSA in the same dyadic context as the dependent variable. The model showed acceptable fit, 𝜒^2^(2, *N* = 52) = 1.96, *p* = 0.376, CFI = 1.00, RMSEA = 0.00, SRMR = 0.08, and the null hypothesis of close fit was retained (*p* = 0.476). The independent variables accounted for 27.0% of the variance in children's RSA. The direct effect of parenting stress was not significant, but the direct effect of task‐assessed EC was significant (*β* = 0.33, *SE* = 0.10, *p* = 0.012) with the interaction term included in the model. The interaction between children's task‐assessed EC and parenting stress was significantly associated with children's RSA during the parent–child interaction tasks (*β* = 0.35, *SE* = 0.11, *p* = 0.006). As shown in Model 2a in Figure 2, parenting stress was negatively associated with child RSA when children had below‐average EC. The Johnson–Neyman analysis showed the slope of parenting stress was significant when task‐assessed EC was ≤0.22 *SD* below the mean (44.23% of cases; *β* = −0.23, *SE* = 0.11, *p* = 0.049) but did not reach significance at any value above the mean (see Model 2b in Figure 2).

### 3.2 Parenting Stress and Child Effortful Control Interactions and Their Association With Children's Parasympathetic Regulation During Independent Tasks

The third model (see Model 3 in Figure 1) examined parenting stress, children's parent‐reported EC, and their interaction as independent variables and children's RSA in an independent context—while completing tasks separated from their parent—as the dependent variable. The model yielded poor fit indices, 𝜒^2^(2, *N* = 54) = 5.04, *p* = 0.080, CFI = 0.87, RMSEA = 0.17, SRMR = 0.11. The model did not show any significant parameter estimates, indicating the interaction between parenting stress and parent‐reported EC was not associated with child RSA during the independently completed tasks. Given the poor global fit, the model was re‐tested with all parameters freely estimated (i.e., a saturated model), allowing for a perfect model fit and more reliable coefficients. In the saturated model, the independent variables accounted for 1.8% of the variance in children's RSA. The resulting parameter estimates changed only marginally and remained nonsignificant, lending further support to there being an absence of associations.

**FIGURE 3:**
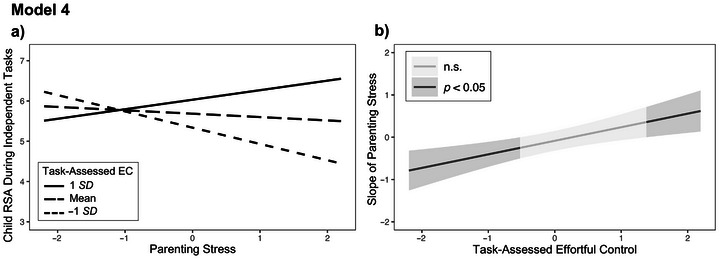
Plotted interactions (a) and regions of significance (b) for the association between parenting stress and children's respiratory sinus arrhythmia (RSA) during independently completed tasks at levels of task‐assessed EC in Model 4. The plotted Johnson–Neyman regions of significance show that the slope of parenting stress was significant when task‐assessed EC was outside the interval [−0.52 *SD*, +1.37 *SD*]. Parenting stress and EC have been standardized for interpretability. n.s. = nonsignificant.

The fourth model (see Model 4 in Figure 1) examined parenting stress, children's task‐assessed EC, and their interaction as independent variables and children's RSA in the same independent context as the dependent variable. The model fit the data well, 𝜒^2^(2, *N* = 54) = 0.26, *p* = 0.878, CFI = 1.00, RMSEA = 0.00, SRMR = 0.04, and the null hypothesis of close fit was retained (*p* = 0.992). The independent variables accounted for 30.6% of the variance in children's RSA. As in the second model, the direct effect of parenting stress was not significant, but the direct effect of task‐assessed EC was significant (*β* = 0.38, *SE* = 0.11, *p* = 0.001) with the interaction term included in the model. The interaction between children's task‐assessed EC and parenting stress was significantly associated with child RSA during the independently completed tasks (*β* = 0.41, *SE* = 0.09, *p* = 0.001). Similar to the first model, parenting stress was negatively associated with child RSA for children with lower EC and positively associated with child RSA for children with higher EC (see Model 4a in Figure 3). The Johnson–Neyman analysis showed the slope of parenting stress was significant when task‐assessed EC was ≤0.52 *SD* below the mean (29.09% of cases; *β* = −0.25, *SE* = 0.12, *p* = 0.049) and ≥1.37 *SD* above the mean (3.67% of cases; *β* = 0.36, *SE* = 0.18, *p* = 0.050; see Model 4b in Figure 3).^3^

## 4 Discussion

The purpose of our study was to build upon progress made in understanding the integration of cognitive and physiological self‐regulatory processes by examining whether preschool‐age children's EC moderates the association between parenting stress and children's parasympathetic regulation. In an effort to foster greater precision in our investigation, both parent‐reported and task‐assessed EC were tested as moderators to probe measurement‐specific associations. Similarly, context‐specificity was tested by exploring differences in associations based on whether children's RSA was assessed during dyadic parent–child tasks or tasks completed independently, separate from their parent. Multiple forms of evidence indicate measurement approach and context play a role in the associations between parenting stress and children's cognitive and physiological self‐regulatory processes. Consistent with prior studies, parents who reported greater stress in their parental role also reported worse EC in their child (e.g., Wagner et al. 2016), but parenting stress was not linearly related to children's performance on the EC task battery. Also, in alignment with past research (e.g., Holzman and Bridgett 2017), children's EC was positively linked to their parasympathetic activity, but associations varied depending on the EC measure and the context of the RSA assessment. Children with better parent‐reported EC did not show a linear relation to their RSA during the independent or dyadic context, while children with better task‐assessed EC had higher RSA across both dyadic and independent contexts. Parenting stress also showed context‐specific associations with child RSA. That is, parenting stress was negatively related to children's RSA during dyadic tasks but was not linearly related to children's RSA during tasks completed independently.

As hypothesized, parenting stress was differentially associated with children's parasympathetic regulation as a function of their EC, with unique associations across EC measures and the context of RSA assessments. Children's parent‐reported EC moderated the association between parenting stress and child RSA only during dyadic tasks, while children's task‐assessed EC served as a moderator of the association between parenting stress and child RSA during both dyadic and independent tasks. The parent‐reported EC moderation effect was specific to the dyadic context, where an increase in parenting stress was associated with higher RSA for children with better parent‐reported EC and lower RSA for those with worse parent‐reported EC. In contrast, the task‐assessed EC moderation effect was present in both contexts. However, the slope and significance of parenting stress at different levels of task‐assessed EC showed distinct patterns. In the dyadic context, higher parenting stress was associated with lower RSA for children with below‐average task‐assessed EC, but there was no effect for children with better EC, contrary to the parent‐report measure. Interestingly, the same pattern observed with the parent‐report measure in the dyadic context was instead found in the independent context: an increase in parenting stress was associated with higher RSA for children with better task‐assessed EC and lower RSA for those with worse task‐assessed EC.

While we expected parenting stress would be negatively associated with child RSA for children with lower EC, we did not expect the positive association with RSA for children with higher EC. Though unanticipated, the pattern of results aligns with the hierarchical integrative model of self‐regulation (Blair and Ku 2022) and a systems view, suggesting preschool‐age children demonstrate adaptive top‐down regulation of bottom‐up physiological activity when their parent experiences heightened stress in their parental role. From an experiential canalization perspective, children's biology and experiences work in tandem to promote some abilities over others (Blair and Raver 2012). This adaptive process is driven by a confluence of factors that have an influential stake in maximizing functioning within one's anticipated environment. For example, children who have adapted to stressful contexts may favor immediate, as opposed to delayed, rewards due to the more advantageous disposition toward reactivity in unpredictable environments (Ellis et al. 2005; Obradović et al. 2010). These developmental trade‐offs may not necessarily result in optimal outcomes and may even appear maladaptive, but they are functionally important within their immediate context (Cox and Paley 1997). With regard to our findings, differences in children's EC ability may reflect adaptations that suit their early experiences, which in turn have consequences for their physiological regulatory capacity (Obradovic 2016).

One explanation for the congruence across models composed of two distinct EC measures and physiology settings is that a similar process emerges when both cognitive and physiological self‐regulation are assessed under comparable contextual demands (Porges 2007). This interpretation is supported by the stronger ecological affinity between assessments of both facets of children's self‐regulation. Relatedly, Darling et al. (2022) found that children's RSA predicted their socially competent behavior only when both were assessed during the same, either structured or unstructured, classroom activities. Viewing these processes as context specific aligns with Doebel's (2020) critique of viewing executive function as domain‐general components that are uniformly applied across situations. Instead, Doebel (2020) argues that children develop skills in engaging control in specific ways for specific goals based on their experiences within their environmental context. Given that parents are presumably only reporting on the behavior they observe when with their child, parent‐reported EC is likely capturing children's engagement of cognitive regulation during typical activities with their parent. Hence, there may be a greater alignment between children's parent‐reported EC and their RSA when engaging in tasks with their parent because the questionnaire measure narrows the focus to the parent–child context (Taylor et al. 2015). On the other hand, EC assessed through structured lab tasks may be capturing children's ability to engage control in a context that is much less reflective of what children typically encounter in their daily lives and in the absence of their parent (Miller‐Cotto et al. 2022). Therefore, children's task‐assessed EC may align more closely with their RSA when engaging in these tasks because both are simultaneously assessed in the same novel, unfamiliar environment (Hastings et al. 2008; Liew et al. 2011).

The adaptive dynamic between children's top‐down cognitive and bottom‐up physiological processes may become less apparent as contextual similarity decreases (Obradović 2016). Therefore, the different findings observed with task‐assessed EC and child RSA during the dyadic setting may be shedding light on how children's regulatory ability in one context is related to their ability in another, rather than the adaptive process that may occur under similar environmental conditions. However, in the independent setting, parent‐reported EC did not moderate the association between parenting stress and child RSA. This may indicate that children's cognitive regulatory ability in the independent setting plays a role in their physiological functioning across contexts, while their cognitive regulatory ability while engaging with their parent is specifically linked to their physiological functioning within a dyadic (i.e., parent–child) environment.

The moderating effect of children's EC has implications for the possible mechanisms at play between contextual factors and children's self‐regulation development. Parent behaviors (e.g., responsiveness; Ward and Lee 2020) are frequently found to mediate pathways between parent psychological stress and child outcomes (Masarik and Conger 2017). Parents who experience greater stress but still engage in supportive parenting behavior may further explain the positive associations observed for children with higher EC. That is, parenting behavior may act as a protective factor (Brody et al. 2019), which enhances children's capacity to optimally regulate. For example, Miller et al. (2015) found that the link between mothers’ physiological stress (i.e., sympathetic arousal) and endorsement of harsh parenting practices was diminished for mothers reporting high compassionate love for their preschool‐age child. On the other hand, stressed parents who engage in harsh parenting behavior may further explain the negative associations observed for children with lower EC (Sturge‐Apple et al. 2012).

Finally, autonomic correlates of parenting behaviors have received increased attention (Choe et al. 2023), as parent physiology may underlie aspects of parent behavior and emotional expression. Given the bidirectionality between parent and child emotions and behavior (Paschall and Mastergeorge 2016), it is important to consider child physiology in connection with parent physiology. For instance, physiological synchrony refers to concordant (i.e., positive synchrony) or discordant (i.e., negative synchrony) patterns of arousal between a parent and their child (Depasquale 2020). Autonomic synchrony has been observed in parents and preschool‐age children during joint tasks, which similarly varies as a function of the task demands and psychopathology risk factors (e.g., maternal aggression; Lunkenheimer, Tiberio, et al. 2018). Further, maltreating mothers and preschool‐age children have shown synchronous RSA suppression during joint tasks (Lunkenheimer, Busuito, et al. 2018). If individuals experiencing heightened stress in their parental role show reduced parasympathetic activity when engaging with their child, children may exhibit a similar parasympathetic response within that context due to autonomic synchrony (Wass et al. 2019). However, the extent to which parents’ autonomic arousal transfers to children's parasympathetic regulation in contexts without their parent is not yet clear. The similarities and differences we observed in EC moderation patterns across the dyadic and independent contexts underscore the need to explore the potential common mechanisms or distinct associations when incorporating parenting behavior and physiology.

### 4.1 Strengths and Limitations

There are several noteworthy strengths of the current study. Our investigation addressed the scarcity of research on the association between parenting stress and children's cognitive and physiological self‐regulation, differences in children's parasympathetic regulation while completing tasks independently compared to with their parent, and unique links to distinct EC measures. We utilized a multimethod approach for greater precision in understanding the complexity of contextual factors and interactivity between self‐regulatory systems. This approach was informed by systems theory (Sameroff and Mackenzie 2003), polyvagal theory (Porges 2007), research on precursory co‐regulation (Buhler‐Wassmann and Hibel 2021), and an application of the recently proposed hierarchical integrative model of self‐regulation (Blair and Ku 2022). Several models with different measures of child EC and settings of physiological assessments provided evidence that parenting stress was differentially associated with children's physiological self‐regulation as a function of their cognitive self‐regulation. The replication of results found within the study strengthens the assertion that cognitive and physiological processes are adaptively integrated and linked to children's immediate context (Frick 2025; Skowron et al. 2014). Interactive effects between parenting stress and children's EC also replicated similar findings of cognitive self‐regulatory processes moderating associations between parent‐related stressors and aspects of children's physiology (Kryski et al. 2013). Last, we found evidence of differences in EC measures extending to biomarkers of children's physiological self‐regulation, lending support to EC measures reflecting true differences in children's skills across settings and contextual demands (Toplak et al. 2013).

Although the study advances the literature in several ways, the findings should be interpreted with consideration of some limitations. First, the cross‐sectional data do not provide conclusive insight into the temporal dynamic among study variables, but parenting stress was reported retrospectively. Therefore, parenting stress is an appropriate independent variable for the current models, and the child variables collected at the visit are unlikely to be causal predictors unless they represent an enduring trait. Second, both EC measures (i.e., parent‐reported and task‐assessed) showed relatively modest internal consistency, which may impact the reliability of study results. Relatedly, the ceiling effects evident in the EC task battery may have contributed to the reduced number of cases in the upper region of significance in the fourth model, with child RSA during dyadic tasks. Third, our relatively small sample is representative of the local area (e.g., high education and income), which may limit generalizability to more diverse samples. These characteristics may explain the lack of significant associations between sociodemographic indicators and the study variables of interest. Last, our measures of children's RSA reflected averages across multiple tasks within each context. While this approach enhanced the reliability of capturing children's general parasympathetic activity in each context, it did not allow for the temporal resolution needed to examine the complexity of children's nonlinear physiological processes during each task.

### 4.2 Future Directions

An important next step is to examine multiple aspects of children's physiology, such as sympathetic and parasympathetic co‐activation, to better understand the similarities and differences in how children dynamically regulate across different settings. Future longitudinal research can provide insight into whether contextual differences become more or less evident over time and into the mechanisms that may be driving developmental change. One question that arises from our results is whether differences in children's parasympathetic regulation from dyadic to independent contexts can be attributed to parents’ own physiology and parent–child physiological synchrony (Lunkenheimer et al. 2015). Investigating this longitudinally may uncover sensitive periods wherein parenting stress and parents’ autonomic activity are most likely to become embedded in children's self‐regulatory abilities. For example, dysfunction within the parent–child relationship that takes place prior to children's normative shift from co‐regulating to self‐regulating could be more impactful for children's parasympathetic regulation (Ostlund et al. 2017). Further, differences in children's parasympathetic regulation across dyadic and independent contexts may not necessarily be attributed to physiological synchrony with their caregiver in the moment (Helm et al. 2018). Rather, earlier calibration of children's physiological regulatory systems from their family environment may be the driver of contextual differences (DePasquale 2020). If a temporal order is established, additional work will be necessary to uncover the role of parenting behaviors in undermining or supporting children's ability to self‐regulate optimally (Suveg et al. 2016). Examining these mechanisms longitudinally is essential to better understand how parenting stress shapes children's environments and adaptations, and whether children's adaptive processes evolve with shifts in children's contextual demands, such as entering school.

## 5 Conclusion

Overall, our results shed light on the complex associations between parenting stress and multiple aspects of preschool‐age children's self‐regulation in environments within and beyond the dyad. These findings lend support to viewing parenting stress as a key factor in children's development of self‐regulation. In the United States, parental stress is a growing public health concern, with 33% of parents reporting high stress levels and 48% feeling completely overwhelmed by their stress in 2023 (Office of the Surgeon General 2024). Parental stressors arise from many sources, from downstream effects of macro‐level changes within the sociopolitical and economic landscape (Smock and Schwartz 2020) to navigating shifts in family composition and stability (Raley and Sweeney 2020). Thus, examining stress derived from a parental role in tandem with children's developmental processes has broad applicability in developing policy measures that support families facing diverse challenges (Anderer 2024). As policymakers, practitioners, and educators continue to prioritize programs aimed at improving cognitive regulatory processes in early childhood (Bailey and Jones 2019; Diamond and Lee 2011; Moffitt et al. 2011), there is increasing recognition of the value of asset‐based approaches (Ellis et al. 2017) and culturally responsive perspectives (Miller‐Cotto et al. 2022). This study contributes to this discourse and reinforces the need to examine associations between parenting stress and both cognitive and physiological facets of children's self‐regulation as contextually dependent and adaptive.

The nuance offered by considering measurement and context specificity underscores the importance of moving beyond considering *what* promotes optimal adaptation to also considering *when*, or in which circumstances, adaptive processes unfold. According to Masten and Barnes (2018), resilience, from a developmental systems perspective, reflects “all the adaptive capacity available at a given time in a given context that can be drawn upon to respond to current or future challenges… through many different processes and connections” (2). Further investigations that acknowledge the adaptive interactivity among self‐regulatory systems are crucial for advancing our understanding of what supports or hinders the development of children's self‐regulation in stressful contexts and for informing intervention and prevention efforts aimed at supporting families experiencing increasing amounts of stress (APA 2023).

## Conflicts of Interest

The authors declare no conflicts of interest.

## Supporting information




**Supplemental Figure 1** Each child's plotted pattern of respiratory sinus arrythmia (RSA) change across the four dyadic parent–child interaction (PCI) tasks (left) and the eight effortful control tasks the child completed independently, separate from their parent (right).

**Supporting Table:** dev70059‐sup‐0002‐tableS1.docx

## Data Availability

The data that support the findings of this study are available from the corresponding author upon reasonable request.

## Endnotes

1 Three parents participated twice when they returned to participate with their younger child who aged into our study. The reported sociodemographic values exclude data from their second visit.

2 The three younger siblings who aged into the study were accounted for in post hoc sensitivity analyses. Each path model was retested with these siblings excluded, which showed negligible differences in the estimates and their significance. To maximize sample size and avoid introducing nonrandom missingness, all results are reported with the siblings included.

3 Post hoc power analyses were conducted for each path model given the relatively small sample size. The first, second, and fourth models yielded greater than 90% power to detect their respective observed effect sizes at a 0.05 significance level, while the third model yielded less than 10% power. It is important to note the fallacy of post hoc power analyses, where statistical power is overestimated with significant observed effects and underestimated with nonsignificant observed effects (Hoenig and Heisey 2001).
