# Supplementary figures and images for: Child Effortful Control Moderates the Link Between Parenting Stress and Child Parasympathetic Regulation: Interactions Across Contexts and Measures
Source: Dev Psychobiol. 2025 Jun 26;67(4):e70059. doi: 10.1002/dev.70059 (PMC12202852; doi:10.1002/dev.70059)

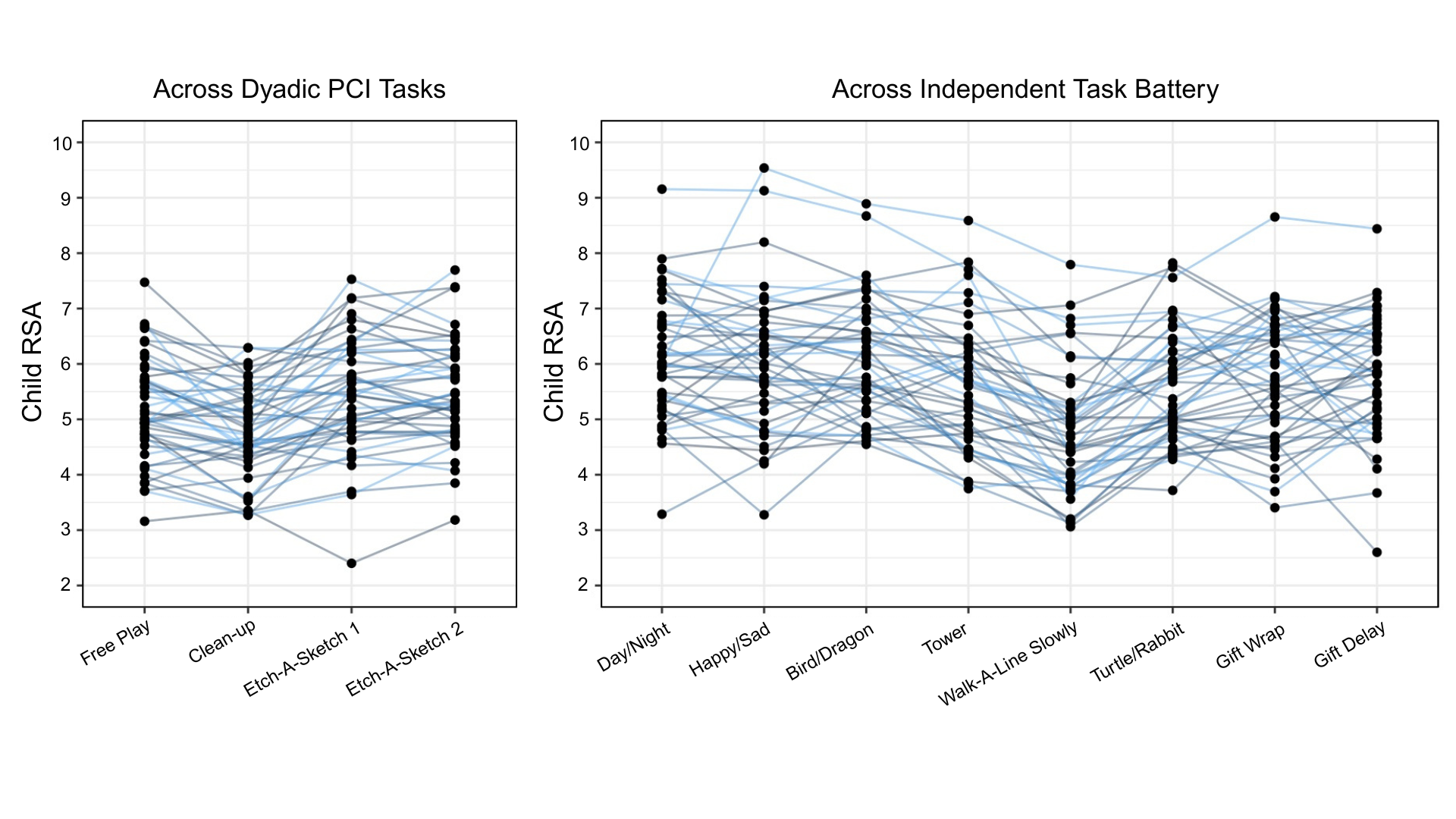

Supplement: Supplementary file 1 — Supplemental Figure 1 Each child's plotted pattern of respiratory sinus arrythmia (RSA) change across the four dyadic parent–child interaction (PCI) tasks (left) and the eight effortful control tasks the child completed independently, separate from their parent (right). [file DEV-67-e70059-s002.png]
