# Supplementary material for: Child Effortful Control Moderates the Link Between Parenting Stress and Child Parasympathetic Regulation: Interactions Across Contexts and Measures
Source: Dev Psychobiol. 2025 Jun 26;67(4):e70059. doi: 10.1002/dev.70059 (PMC12202852; doi:10.1002/dev.70059)
Supplement: Supplementary file 2 — Supporting Table: dev70059‐sup‐0002‐tableS1.docx [file DEV-67-e70059-s001.docx]

**Supplemental Table 1 |** Fit Statistics of Four Alternative Path Models

|  | **𝜒^2^** | ***df*** | ***p*** | **CFI** | **RMSEA** | **SRMR** |
| --- | --- | --- | --- | --- | --- | --- |
| Model 1 | 163.58 | 2 | .000 | 0.15 | 1.25 | .50 |
| Model 2 | 165.69 | 2 | .000 | 0.07 | 1.26 | .71 |
| Model 3 | 153.54 | 2 | .000 | 0.18 | 1.17 | .38 |
| Model 4 | 151.99 | 2 | .000 | 0.06 | 1.17 | .04 |

*Note.* Model 1 examined parenting stress, children’s RSA in a dyadic context (i.e., during parent–child tasks), and their interaction as predictors of children’s parent-reported EC. Model 2 examined parenting stress, children’s RSA in a dyadic context, and their interaction as predictors of children’s task-assessed EC. Model 3 examined parenting stress, children’s RSA in an independent context (i.e., during the EC task battery the child completed while separate from their parent), and their interaction as predictors of children’s parent-reported EC. Model 4 examined parenting stress, children’s RSA in an independent context, and their interaction as predictors of children’s task-assessed EC.
 𝜒^2^  = robust chi-square test of exact fit; CFI = comparative fit index; RMSEA = root mean square error of approximation; SRMR = standardized root mean square residual.
